# Quantitative Coronary Atherosclerotic Plaque Burden From CCTA and the Benefit From Lipid-Lowering Medication

**DOI:** 10.1161/CIRCIMAGING.125.018840

**Published:** 2026-04-06

**Authors:** Teemu Maaniitty, Sarah Bär, Jeroen J. Bax, Juhani Knuuti, Antti Saraste

**Affiliations:** 1 Turku PET Centre, Turku University Hospital and University of Turku, Finland (T.M., S.B., J.K., A.S.).; 2 Department of Clinical Physiology, Nuclear Medicine and PET, Turku University Hospital, Finland (T.M., J.K.).; 3 Department of Cardiology, Bern University Hospital Inselspital, Switzerland (S.B.).; 4 Department of Cardiology, Leiden University Medical Center, the Netherlands (J.J.B.).; 5 Heart Center, Turku University Hospital and University of Turku, Finland (J.J.B., A.S.).; 6 Faculty of Medicine, University of Turku, Finland (J.J.B., J.K., A.S.).

**Keywords:** artificial intelligence, coronary artery disease, lipids, middle aged, prognosis

## Abstract

**BACKGROUND:**

We hypothesized that quantification of coronary atherosclerotic plaque burden by artificial intelligence-guided quantitative computed tomography can identify patients who derive outcome benefit from lipid-lowering medication (LLM).

**METHODS:**

In this observational cohort study, consecutive symptomatic patients undergoing coronary computed tomography angiography for suspected coronary artery disease (CAD) were assessed for percent atheroma volume (PAV) by artificial intelligence-guided quantitative computed tomography. The use of LLM was assessed based on drug purchase registry data within 6 months after coronary computed tomography angiography. Patients were followed for the composite of death, myocardial infarction, and unstable angina for a median of 6.9 years.

**RESULTS:**

Among 2269 patients (median age, 63 years; 42% men), 1261 (56%) patients used LLM after coronary computed tomography angiography, and 255 (11%) experienced the composite end point during follow-up. The median PAV was 6.6% among users and 1.4% among nonusers of LLM (*P*<0.001). Adapting the previously proposed CAD stages for artificial intelligence-guided quantitative computed tomography, the use of LLM (versus no use) was associated with improved outcomes among the 910 patients with PAV >5% (annual event rate, 2.62% versus 4.14%; adjusted *P*=0.002), even in the absence of obstructive CAD, but not among the 1359 patients with PAV ≤5% (annual event rate, 0.94% versus 0.65%; adjusted *P*=0.717). An adjusted Cox regression analysis, including interaction between PAV and LLM, suggested a PAV threshold between 4% and 10% for gaining prognostic benefit from LLM.

**CONCLUSIONS:**

In symptomatic patients with suspected CAD, LLM after coronary computed tomography angiography was associated with a lower rate of adverse events during long-term follow-up among those with PAV >5%, even in the absence of obstructive CAD. The quantification of coronary atherosclerotic plaque burden is a potential marker to guide preventive lipid-lowering therapy.

**Clinical Perspective**

Lipid-lowering medication is recommended in obstructive coronary artery disease to prevent disease progression and clinical adverse events, whereas its role in nonobstructive coronary artery disease is less well established. Recent technological advances have enabled semiautomated quantification of coronary atherosclerotic plaque burden from coronary computed tomography angiography, measured as percent atheroma volume. In this observational cohort study of 2269 symptomatic patients undergoing coronary computed tomography angiography for suspected coronary artery disease, the use of lipid-lowering medication (versus no lipid-lowering medication) after coronary computed tomography angiography was associated with a lower rate of long-term adverse events among those with moderate-to-severe coronary atherosclerosis on coronary computed tomography angiography (defined as percent atheroma volume >5%), even in the absence of obstructive coronary artery disease. The quantification of coronary atherosclerotic plaque burden appears as a potential marker to guide preventive lipid-lowering therapy, but prospective studies are needed to assess its effectiveness and establish clinical decision thresholds across different software tools.

Lipid-lowering medication (LLM) is recommended in obstructive coronary artery disease (CAD) to prevent disease progression and clinical adverse events.^1,2^ The role of LLM in nonobstructive CAD is less well established, although large observational studies have demonstrated beneficial prognostic effects from LLM also in this patient group.^3–6^

Coronary computed tomography angiography (CCTA) is often the first-choice diagnostic test for patients with a low-to-intermediate likelihood of obstructive CAD.^2,7^ While the prevalence of obstructive CAD has declined based on contemporary patient cohorts, nonobstructive CAD is commonly detected by CCTA.^8,9^ Moreover, the majority of clinical events occur among individuals with nonobstructive CAD,^10,11^ and when stratified by the extent of coronary atherosclerosis, measured by coronary artery calcium score (CACS), similar clinical outcomes have been found in obstructive and nonobstructive CAD.^12^ Recent technological advances have made absolute quantification of coronary atherosclerotic plaque burden feasible from CCTA images.^13^ Artificial intelligence-guided quantitative computed tomography (AI-QCT) is one of these tools, with clearance by the US Food and Drug Administration.^14,15^ Increasing coronary plaque burden measured by AI-QCT is associated with worsening long-term outcomes.^16,17^

The aim of this study was to investigate the relationship between coronary atherosclerotic plaque burden, LLM, and long-term clinical outcomes after CCTA in a real-world cohort of symptomatic patients with suspected CAD. We aimed to investigate the association of LLM use after CCTA on the long-term clinical outcomes according to a staging of coronary plaque burden based on percent atheroma volume (PAV).^16,18,19^ Second, we aimed to identify a threshold for quantitative coronary plaque burden associated with an outcome benefit from LLM.

## Materials and Methods

### Patients

From an institutional registry, we identified 2409 consecutive symptomatic patients undergoing CCTA for suspected CAD at Turku University Hospital (Turku, Finland) from 2007 to 2016. Patients undergoing CCTA primarily for reasons other than suspected CAD, such as cardiomyopathy or preoperative evaluation, or with previously known CAD were not considered for inclusion. After excluding 137 patients due to nonretrievable CCTA and 3 with incomplete follow-up, the final study cohort consisted of 2269 patients. The study complies with the Declaration of Helsinki. The ethics committee of the hospital district of Southwest Finland approved the study protocol and waived the need for written informed consent. The data supporting the findings are not publicly available due to privacy and ethical restrictions.

### Imaging Procedures

CCTA scans were performed using 64-row hybrid positron emission tomography–computed tomography scanners (GE Discovery VCT or GE D690; General Electric Medical Systems, Waukesha, WI). Before CCTA, intravenous metoprolol (0–30 mg) and sublingual/oral nitrate were administered. A noncontrast computed tomography scan for measuring Agatston CACS was acquired before CCTA in most patients. Intravenous low-osmolal iodine contrast agents were used for CCTA. Prospective electrocardiography-triggered acquisition during diastole was applied whenever feasible. According to the institutional imaging protocol, patients with a suspected obstructive (visually ≥50% diameter) stenosis on CCTA underwent functional testing with ^15^O-water positron emission tomography myocardial perfusion imaging as previously described.^20^

### Quantitative CCTA Analysis

The CCTA scans were reanalyzed in 2022 to 2023 in a blinded manner by AI-QCT (Cleerly LABS, Cleerly, Inc, Denver, CO), which is a US FDA-cleared software service based on a series of validated convolutional neural networks.^14,15^

Atherosclerotic plaque volume was calculated for each coronary artery segment and summed on a patient level. Plaque volume was then normalized to the vessel volume of the individual coronary artery tree, to obtain percent atheroma volume (PAV = 100×plaque volume [mm^3^]/vessel volume [mm^3^]). In addition, different plaque components were similarly normalized to vessel volume, to obtain percent noncalcified, low-density, and calcified plaque volumes.^16^ Low-density plaque was prevalent in very small quantities and was combined into noncalcified plaque volume (NCPV) in further analyses. The predefined 5% cutoff value for PAV that was utilized in the current study is based on a previously proposed coronary artery plaque staging system, where PAV 0% to 5% indicates no or mild atherosclerotic plaque burden and PAV >5% indicates moderate or severe plaque burden.^16,18,19^ The rationale for using a binary cutoff value (ie, PAV ≤5% versus >5%) instead of the originally proposed 4 stages (ie, PAV 0%, >0% to 5%, >5% to 15%, >15%) was the observation that PAV of 0% was uncommon in our cohort, being consistent with previous literature,^16^ and the very small number of patients with PAV >15% but not using LLM after CCTA. Percent diameter stenosis is reported on a patient level as the highest degree of stenosis in each patient, with ≥50% diameter stenosis considered as obstructive.

For comparison, segment involvement score (SIS) was calculated as a semiquantitative measure of coronary atherosclerotic plaque burden, defined as the number of coronary segments with any atherosclerosis by visual CCTA reading and categorized as ≤4 versus >4.^3^ In addition, CACS by the Agatston method was available in 1862 (82% of 2269) patients. Standard clinical reporting of CCTA ± CACS ± positron emission tomography perfusion was available to guide patient management,^20^ whereas AI-QCT analysis was performed post hoc and did not impact the treatment decisions.

### Lipid-Lowering Medication

The use of LLM was assessed based on a national database of drug purchases provided by the Social Insurance Institution of Finland. The use of LLM before CCTA was defined for each individual patient as the presence of ≥1 purchase of statin or ezetimibe within the 6 months preceding CCTA. Similarly, the use of LLM after CCTA was defined as any purchase of statin or ezetimibe within the 6 months following CCTA. In addition, the use of LLM was assessed at 6 to 12 months before CCTA (to reflect the baseline use of LLM before the diagnostic workup) and at 18 to 24 months after CCTA (to evaluate adherence to the medication). In Finland, LLM is only available from pharmacies with a physician’s prescription and dispensed for a maximum of 3 months’ usage at a time, and all purchases are recorded in the national database. We analyzed purchases of statin and ezetimibe only, as the number of patients using other types of LLM was negligible in our cohort (<10 patients).

### Clinical Data and Outcome

Baseline patient characteristics including symptoms and cardiovascular risk factors were collected from electronic medical records. Risk factor-weighted clinical likelihood of obstructive CAD was calculated according to the 2024 European Society of Cardiology guidelines for chronic coronary syndromes.^2^ Early myocardial revascularization was defined as a percutaneous coronary intervention or coronary artery bypass graft surgery within 6 months after CCTA. Long-term follow-up data on all-cause death and acute coronary syndrome, including myocardial infarction (MI) and unstable angina pectoris, were derived from hospital discharge registry data (Auria Clinical Informatics) until May 2020. Diagnoses of acute coronary syndrome were individually verified against electronic medical records.

### Statistical Analyses

Continuous variables are presented as median (25th–75th percentile) and compared using the independent-samples Mann-Whitney *U* test (nonnormal distribution). Categorical variables are presented as counts and percentages and compared using the χ^2^ test. Spearman rank correlation was assessed between CACS and PAV. The composite end point was defined as an all-cause death, MI, or unstable angina pectoris, whichever occurred first. The annual (crude) rate of the composite end point with 95% CIs was estimated by Poisson regression, based on the number of adverse events divided by person-time of follow-up, and expressed as a percentage. Kaplan-Meier survival curves were created and compared using the log-rank test.

Univariable and multivariable Cox regression analyses were performed for the composite end point, including LLM after CCTA (binary variable) and PAV or NCPV (continuous variables) as main effects as well as their interaction. Cox multivariable regression included all clinical variables (age, sex, cardiovascular risk factors and symptoms) and early revascularization as covariates. Analyses stratified by binary PAV or NCPV (using the 5% cutoff value) were additionally adjusted for CACS, which was first log-transformed and nonexisting values (18%) were imputed (20 data sets) using fully conditional specification with predictive mean matching (5 donors). The imputation model included all Cox model covariates, event indicator, and time-to-event, and the results from each imputed data set were pooled using Rubin’s rules. As an alternative to multivariable adjustment, we created a propensity score based on all data available before CCTA (ie, age, sex, cardiovascular risk factors, symptoms, and medication before CCTA [Table S1]) using a multivariable binary logistic regression model that predicts the use of LLM within 6 months after CCTA. Subsequently, Cox regressions were performed adjusting for this propensity score (and early revascularization). Adjusted hazard ratio (HR) with 95% CI were estimated by the multivariable Cox regression including interaction and graphically presented across the values of PAV or NCPV using the *plotINT* function of the *interactionRCS* package in R.

Two-sided *P*<0.05 was considered statistically significant. The statistical analyses were conducted using IBM SPSS Statistics, version 27, and R, version 4.3.2.

## Results

### Coronary Plaque Burden and Use of LLM

The cohort of 2269 patients is described in Table 1. The median age was 63 years, 42% were men, and 23% had typical angina pectoris. Cardiovascular risk factors were common, and 45% were using LLM before CCTA. Obstructive CAD (≥50% diameter stenosis) was revealed by visual CCTA reading in 31% and by AI-QCT analysis in 24% of patients. According to local practice, 920 patients underwent downstream stress positron emission tomography myocardial perfusion imaging, showing ischemia in 411 (45%) patients. A total of 213 patients underwent early revascularization, most of which (n=164, 77%) occurred among those with positron emission tomography-detected ischemia. CACS was measured in 1862 (82%) patients with a median of 37 (25th–75th percentiles, 0–259) and strongly correlated with PAV (Spearman ρ=0.894, *P*<0.001).

**Table 1.**
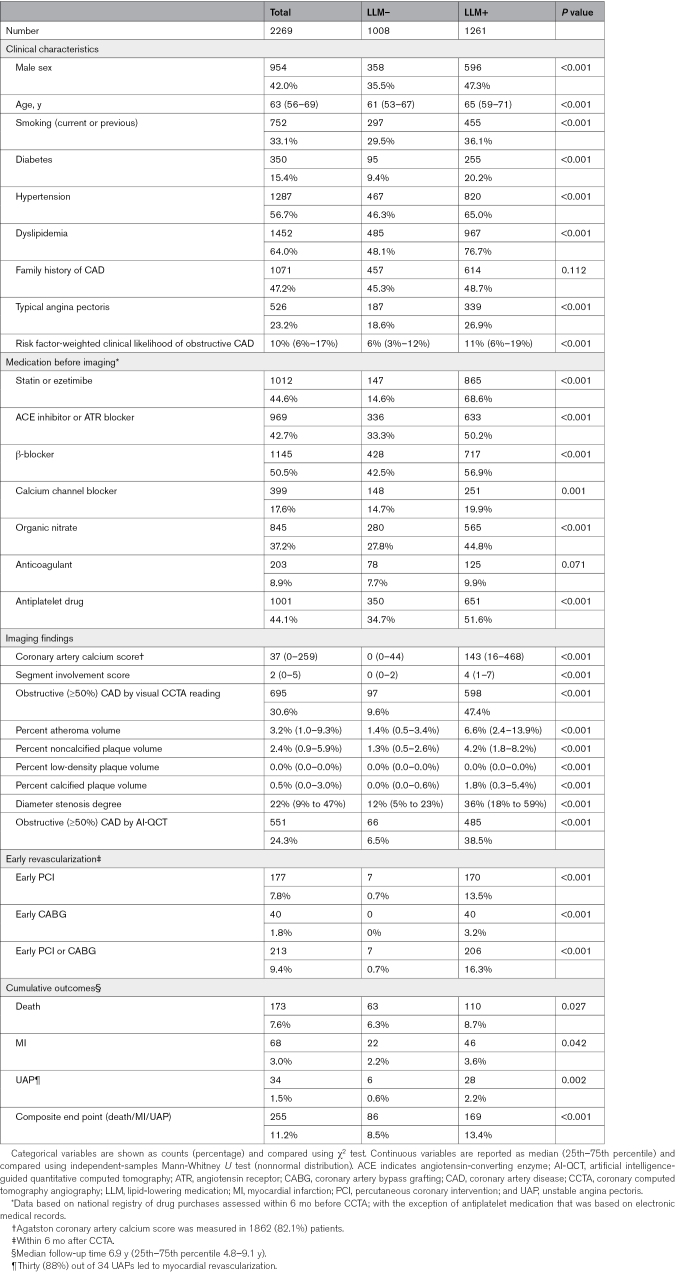
Patient Characteristics. Patient Characteristics in Total Cohort and Stratified by the Use of Lipid-Lowering Medication Within 6 Months After CCTA

**Figure 1.**
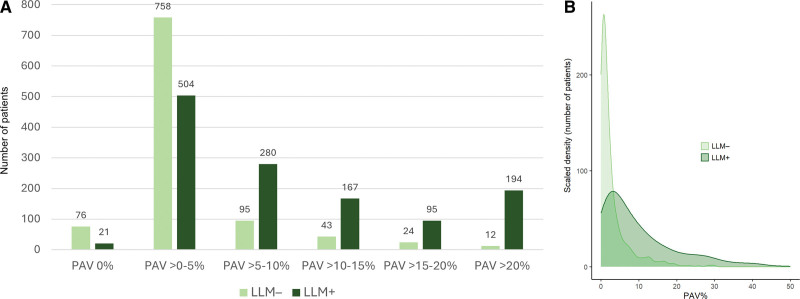
**Plaque burden and use of lipid-lowering medication.** Numbers of patients using lipid-lowering medication (LLM+) and not using (LLM–) as assessed within 6 months after coronary computed tomography angiography (CCTA), stratified by 5% increments of percent atheroma volume (PAV; **A**) and shown as continuous density curves scaled to reflect the number of patients in each group (**B**).

Based on drug purchase data, 1261 (56%) patients were users of LLM as assessed within 6 months after CCTA. Of LLM users, 97.9% were using statin, 0.9% ezetimibe, and 1.2% both. The users of LLM, as compared with nonusers, were older, more often men, had more cardiovascular risk factors and medications, higher clinical likelihood of obstructive CAD, had more advanced coronary atherosclerosis, and more often underwent early revascularization after CCTA (Table 1). Only 63 (13%) of 497 patients with very low (≤5%) clinical likelihood of obstructive CAD had PAV >5%, whereas 354 (38%) of 933 patients with low (>5% to 15%) clinical likelihood had PAV >5%. The median PAV was 6.6% among users and 1.4% among nonusers of LLM (*P*<0.001). Figure 1 shows the increasing relative proportion of LLM users with increasing PAV.

Among the 1261 patients using LLM after CCTA, 576 (46%) patients were using LLM at baseline (ie, 6–12 months before CCTA), whereas 685 (54%) were new users. Consistently, among the 736 patients with PAV >5%, the proportion of new LLM users was 55%. Discontinuation of LLM after CCTA among patients who were using it before imaging was uncommon (Table S2). However, among the 1261 patients who were using LLM within 6 months after CCTA, 276 (22%) had discontinued using LLM at 18 to 24 months after CCTA. In turn, of the 1008 patients not using LLM within 6 months after CCTA, 80 (8%) had started using LLM at 18 to 24 months (Figure S1).

### Clinical Outcome According to LLM, Stages of Plaque Burden and Obstructive CAD

The follow-up time was a median of 6.9 years (25th–75th percentiles, 4.8–9.1 years), during which 255 (11%) patients experienced the composite end point of death, MI, or unstable angina pectoris. The cumulative incidence of the composite end point and all of its components was higher among users than nonusers of LLM (composite end point, 13.4% versus 8.5%, *P*<0.001; Table 1).

**Figure 2.**
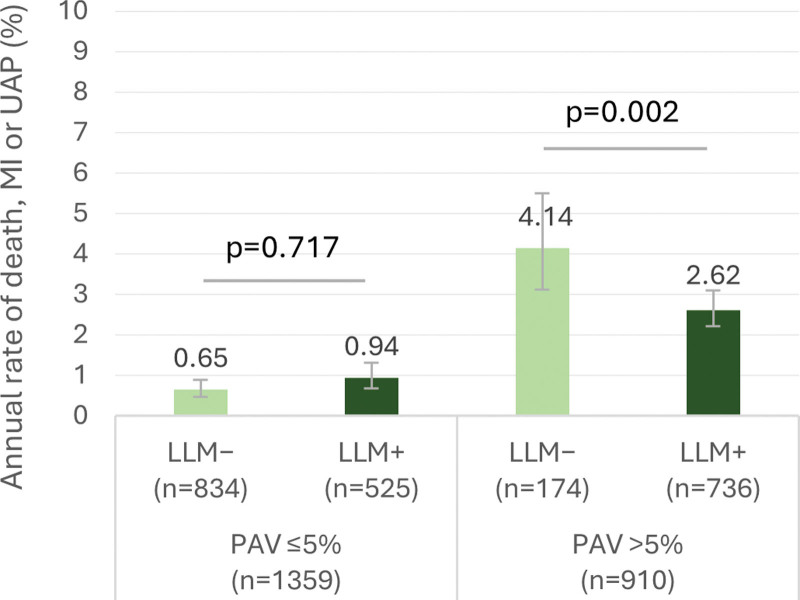
**Lipid-lowering medication and clinical outcome according to stages of percent atheroma volume (PAV).** Annual (crude) incidence of death, myocardial infarction (MI), or unstable angina pectoris (UAP) in patients using lipid-lowering medication (LLM+) vs not using (LLM−) as assessed within 6 months after coronary computed tomography angiography (CCTA), categorized by a predefined PAV cutoff (≤5% vs >5%). Error bars represent 95% CIs. *P* values are based on multivariable Cox regression adjusted for age, sex, smoking, diabetes, hypertension, dyslipidemia, family history, presence of typical angina, coronary artery calcium score, and early revascularization.

Patient characteristics stratified by PAV (≤5% versus >5%) are shown in Table S3. The cumulative incidence of the composite end point and its components was higher in 910 patients with PAV >5% than in 1359 patients with PAV ≤5% (composite end point 19.9% versus 5.4%, death 12.6% versus 4.3%, MI 6.0% versus 1.0%, unstable angina pectoris 3.1% versus 0.4%; all *P*<0.001). Among patients with PAV ≤5%, users and nonusers of LLM within 6 months after CCTA had comparably low annual rates of the composite end point (0.94% versus 0.65%, respectively; multivariable-adjusted HR, 0.91 [95% CI, 0.54–1.52], *P*=0.717; propensity score adjusted HR, 0.90 [95% CI, 0.50–1.59]; *P*=0.707). In contrast, among patients with PAV >5%, the users of LLM had a significantly lower annual adverse event rate as compared with the nonusers (2.62% versus 4.14%, risk reduction 37%; multivariable-adjusted HR, 0.57 [95% CI, 0.40–0.82], *P*=0.002; propensity score adjusted HR, 0.54 [95% CI, 0.37–0.78]; *P*=0.001), despite a slightly higher PAV level (median PAV, 12.1% versus 9.0%; *P*<0.001; Figure 2). For comparison, patients with SIS >4 by visual CCTA reading and CACS >100 showed similar trends of risk reduction with LLM but not reaching statistical significance (SIS>4: adjusted HR, 0.70 [95% CI, 0.46–1.07], *P*=0.101; and CACS >100: HR, 0.77 [95% CI, 0.50–1.19], *P*=0.242; Figure S2). Consistent with the findings for PAV, there was a significant association between LLM use and lower event rates among patients with NCPV >5% (adjusted HR, 0.64 [95% CI, 0.42–0.96], *P*=0.033; Figure S3).

**Figure 3.**
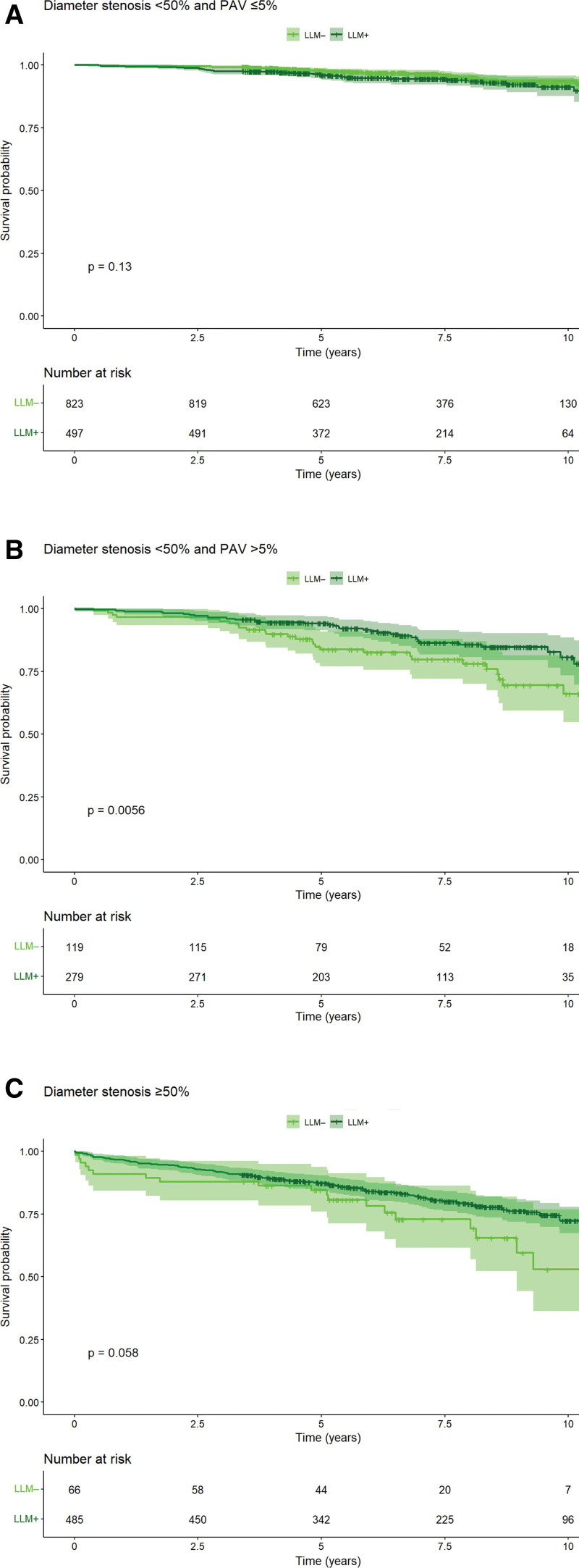
**Lipid-lowering medication and long-term clinical outcome according to obstructive coronary artery disease (CAD) and percent atheroma volume (PAV).** Kaplan-Meier curves (with 95% CIs and log-rank *P* values) for survival free from the composite end point (death, myocardial infarction, unstable angina pectoris) for patients using lipid-lowering medication (LLM+) vs not using (LLM−) as assessed within 6 months after coronary computed tomography angiography (CCTA). Survival curves are shown separately for the subgroups of diameter stenosis <50% and PAV ≤5% (**A**), diameter stenosis <50% and PAV >5% (**B**), and diameter stenosis ≥50% (**C**).

Patients with obstructive CAD (≥50% diameter stenosis) had a higher rate of the composite end point than patients without obstructive CAD (22.1% versus 7.7%, *P*<0.001). In the absence of obstructive CAD, use of LLM was associated with better long-term event-free survival in patients with PAV >5% but not in those with PAV ≤5% (Figure 3). In obstructive CAD, only 66 (12%) patients were not using LLM, and the beneficial association of LLM with survival was borderline significant (log-rank *P*=0.058). Moreover, 93% of patients with obstructive CAD had PAV >5%, precluding further subgroup analysis according to PAV.

### Interaction of Plaque Burden and LLM

**Figure 4.**
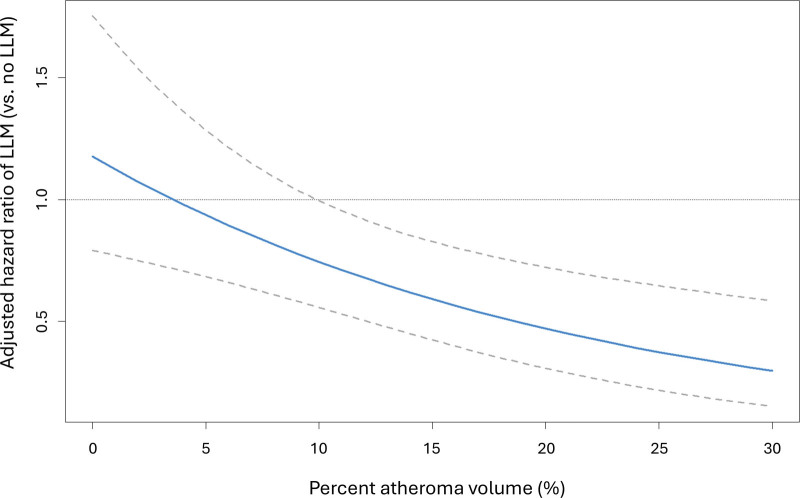
**Interaction of percent atheroma volume (PAV) and lipid-lowering medication (LLM) in terms of long-term clinical outcome.** Cox regression model for the interaction of continuous PAV (%) and use of LLM assessed within 6 months after coronary computed tomography angiography (CCTA), for the composite end point of death, myocardial infarction, or unstable angina pectoris. The model is multivariable adjusted for age, sex, smoking, diabetes, hypertension, dyslipidemia, family history, presence of typical angina, and early revascularization. The solid line represents the estimate of adjusted hazard ratio (HR) of the use of LLM (vs no LLM), with dashed lines showing 95% CIs. The HR estimate equals to 1 (ie, no effect by LLM) at PAV of ≈4%, and the upper 95% confidence limit equals to 1 at PAV of ≈10%.

There was a significant interaction between PAV and LLM in terms of the composite end point (*P*-interaction=0.002; Table 2). This is graphically presented in Figure 4, showing the adjusted HR related to the use of LLM (versus no LLM) after CCTA across the values of PAV. The HR estimate equals 1 (ie, no effect of LLM) at a PAV of ≈4%, whereas the upper 95% confidence limit of the HR intersects the line of equality at a PAV of ≈10%. For comparison, analysis of noncalcified plaque burden suggested a threshold of 3% to 6% for percent NCPV to gain prognostic benefit from LLM (Figure S4; Table S4). Of note, there was no interaction between CACS and LLM in relation to outcome (adjusted *P*-interaction=0.254; n=1862).

**Table 2.**
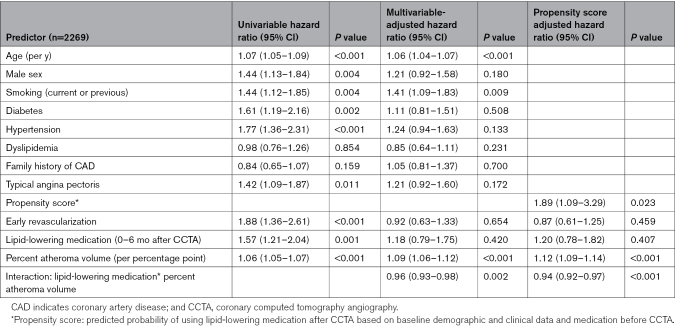
Predictors of Long-Term Outcome. Univariable, Multivariable, and Propensity Score Adjusted Cox Regressions for Predicting the Composite End Point (All-Cause Death, Myocardial Infarction, Unstable Angina Pectoris), Including Interaction of Percent Atheroma Volume (Continuous Variable) and Use of Lipid-Lowering Medication as Assessed Within 6 Months After CCTA

## Discussion

We studied the relationship between quantitatively measured coronary atherosclerotic plaque burden and LLM use after CCTA in predicting long-term clinical outcomes in a real-world cohort of symptomatic patients with suspected CAD. Using a predefined PAV cutoff value of 5%, we found that the users of LLM—assessed within 6 months after CCTA—as compared with nonusers had a significantly lower annual rate of death or acute coronary syndrome in the context of moderate-to-severe coronary plaque burden (PAV >5%), whereas patients with no or mild plaque burden (PAV ≤5%) had comparably low rates of adverse events irrespective of LLM. Importantly, this beneficial association of LLM with outcome was also observed in patients with nonobstructive CAD but PAV >5%. Furthermore, we studied the interaction between continuous PAV and the use of LLM, and found that a PAV threshold between 4% and 10% might be used to identify patients who benefit from LLM after CCTA in terms of long-term outcomes.

Our results are in line with previous observational studies demonstrating the prognostic benefit of LLM with increasing severity of CAD. In the multicenter CONFIRM (Coronary CT Angiography Evaluation For Clinical Outcomes: An International Multicenter) registry, baseline use of statins was associated with a 43% lower adjusted risk of death or acute coronary syndrome in patients with obstructive CAD on CCTA.^21^ In patients with no or nonobstructive CAD, the increasing extent of atherosclerosis measured by CACS or SIS was associated with a stepwise increase in all-cause mortality among patients not using statins, whereas this risk increase was mitigated among those using statins at baseline.^22^ Hulten et al^3^ found that statin medication after CCTA was associated with a reduced rate of cardiovascular death or MI among patients with extensive nonobstructive CAD (SIS >4 and <50% stenosis). Øvrehus et al^6^ studied a real-world cohort of symptomatic patients without obstructive CAD on CCTA and assessed post-CCTA statin therapy based on national registry data, similar to our study. The authors found that the use of statins was associated with a reduced rate of all-cause mortality and MI, with increasing absolute benefit with increasing coronary artery calcium burden. Moreover, our results agree with the randomized SCOT-HEART (Scottish Computed Tomography of the Heart) trial showing that CCTA-guided patient management involves more frequent use of preventive medical therapy and associates with improved long-term clinical outcomes.^23^ Furthermore, it has been shown by serial intracoronary imaging that high-intensity LLM induces favorable changes in nonobstructive coronary plaques after acute MI, and this is associated with a lower short-term risk of adverse cardiovascular events.^24^

Our study extends the evidence to the use of AI-QCT for quantification of coronary atherosclerotic plaque burden from CCTA and its association with prognostic benefit from LLM. The PAV reflects total atherosclerotic plaque volume across the coronary tree, normalized by the vessel volume to facilitate interindividual comparisons.^25^ This is in contrast to semiquantitative measures, such as SIS, which are surrogate markers of the extent of coronary atherosclerosis, but do not measure within-segment plaque burden. In the current study, we found a similar trend of LLM benefit among those with SIS >4 by visual CCTA reading or CACS >100 as in those with PAV >5% by AI-QCT; however, not reaching statistical significance after multivariable adjustment. CACS is widely used in clinical practice and was available to guide patient management in our cohort. However, after adjustment for CACS, we observed a significant association between LLM and outcome among patients with elevated total (PAV >5%) or noncalcified plaque burden (NCPV >5%). In contrast, we observed no significant interaction between CACS and lipid-lowering therapy in relation to long-term outcomes. These results suggest that quantitative plaque burden may provide information beyond CACS when identifying patients who derive prognostic benefit from LLM. Although PAV was strongly correlated with CACS, PAV is biologically distinct from CACS, which only measures calcified plaque components, and a recent study demonstrated superior discriminatory power of PAV as compared with CACS in predicting long-term mortality or MI.^26^ Importantly, statins have a confounding effect on CACS, as they may promote the transformation of coronary atherosclerosis toward more calcified components, but still reduce the risk of disease progression.^27,28^ A prospective randomized study would be needed to formally investigate the effect of quantitative CCTA analysis on long-term clinical outcomes. Moreover, we focused on total plaque burden rather than different plaque components, an approach supported by a previous prognostic study^17^ and potentially making the results more generalizable across different quantification software tools.

Previously, 4 stages (0–3) of coronary atherosclerosis have been proposed for prognostic classification of patients based on PAV measured by AI-QCT: PAV 0%, >0% to 5%, >5% to 15%, and >15%.^16,18,19^ However, in the current study, we used a simplified approach based on a binary cutoff value of 5% for PAV. This was justified by the fact that the absence of plaque (ie, PAV 0%) was rare in our symptomatic cohort with suspected CAD (n=97, 4%), consistent with a previous report.^16^ Similarly, the number of patients with PAV >15% (stage 3 CAD) but not using LLM after CCTA was very low (n=36), precluding individual outcome analyses in these subgroups.

As the second objective, we studied PAV as a continuous measure using a multivariable-adjusted Cox regression model with interaction. We identified PAV ≈4% as a point beyond which LLM was associated with a long-term outcome benefit (HR estimate <1). The upper 95% confidence limit of HR intersected the line of equality at a PAV of ≈10%, indicating a definite, statistically significant association with PAV >10% in this cohort. This wide range (4% to 10%) may partly reflect the continuous nature of risk and partly statistical uncertainty, as the CIs for HR are directly affected by the study sample size. While our study is, to our knowledge the first to explore the interaction between LLM and coronary plaque burden quantified by AI-QCT, the relatively large CIs observed in our study highlight the need for future larger-scale and prospective studies to establish thresholds across different software tools and give recommendations for clinical decision-making. Interestingly, the predefined binary prognostic PAV cutoff point of 5% falls within the range where our Cox interaction model suggested a threshold for gaining prognostic benefit from LLM, that is, between 4% and 10%.

The findings of our study support the use of LLM as a preventive medication, particularly in those with moderate-to-severe coronary atherosclerosis (ie, PAV >5%), and importantly, even in the absence of obstructive coronary stenosis. This is in line with the previous observations among individuals with nonobstructive CAD.^3,6,22^ Our findings in patients with obstructive CAD (≥50% diameter stenosis) should be interpreted with caution because of the small number (n=66) of patients not using LLM in this subgroup, which reflects the established role of LLM in obstructive CAD.^1,2^

Patients with no atherosclerosis or mild atherosclerotic plaque burden (ie, PAV 0% to 5%) constitute 60% of our study cohort and had generally favorable outcomes, with annual adverse event rates <1%. There was no outcome difference between the users and nonusers of LLM in those with PAV 0% to 5%, and the CIs of HR by the Cox interaction model were wide in the lower range of PAV. However, due to our observational study design, even after statistical multivariable adjustments, we cannot rule out potential prognostically beneficial effects of LLM among individuals with a limited amount of coronary atherosclerosis such as PAV ≤5%, especially beyond the median follow-up of 6.9 years.

The use of comprehensive national registry data on drug purchases in the current study better reflects the actual medication use than drug prescription data or self-reporting by patients. Moreover, we observed LLM purchases (being predominantly statins) within 6 months after the CCTA imaging, in contrast with some previous studies utilizing baseline medication data before CCTA.^21,22^ This is important because we and others have previously shown that CCTA findings significantly impact the use of LLM.^20,29^ Finally, our study reflects the use of LLM in a real-world context where patient management is often suboptimal.^30^

### Limitations

Our study was observational, and residual confounding is possible, although the analyses were adjusted for potential confounders such as early myocardial revascularization. Moreover, results of multivariable-adjusted analyses and propensity score adjusted analyses were consistent. However, residual confounding may exist, for example, related to nonrecorded patient characteristics and clinical decision-making factors that cannot be fully captured in registry-based data.

LLM could not be used as a time-varying factor, as medication status was not available for the total long-term follow-up. However, we found that within 2 years after CCTA it was more common to stop LLM than to start LLM, and therefore, our definition of LLM use (ie, within 6 months after CCTA) more likely leads to underestimation rather than overestimation of the prognostic benefit from LLM. Intensity of lipid-lowering therapy was not considered, because the dosage of LLM was not available from the national registry. Drug purchase data for aspirin were not available, as it is a prescription-free drug in Finland. A major limitation of our study is that plasma lipid levels were not available. The CCTA scans were performed in the era before novel LLM agents such as proprotein convertase subtilisin/kexin type 9 inhibitors. Moreover, the CCTA scans were performed with 64-detector row scanners, whereas newer scanner technology might improve image quality.

We acknowledge that using a binary cutoff for coronary plaque burden (such as PAV 5%), although easily applicable for clinical use, may be a too simplistic approach; therefore, we also studied PAV as a continuous variable. Plaque quantification results are not necessarily concordant between different software,^31^ and we cannot evaluate whether similar cutoffs are applicable to analysis tools other than AI-QCT. To the best of our knowledge, no studies have been published evaluating the impact of scanner and acquisition protocols on the quantitative plaque volumes by AI-QCT. However, previous studies—using invasive quantitative coronary angiography as a reference standard—have demonstrated that AI-QCT outperforms clinical CCTA reading in assessing stenosis degree^32^ and that the diagnostic performance of AI-QCT in detecting obstructive ≥50% stenosis is unaffected by commonly used CCTA scanning parameters and scanners.^33^

Considering these limitations, our current study should trigger larger-scale observational studies utilizing quantification of coronary plaque burden and comprehensive reliable medication data. Subsequently, the imaging-guided approach to the treatment of CAD, such as targeting novel effective lipid-lowering agents, should be tested in prospective, randomized study settings.^34^ It is important to note that AI-QCT analysis was performed post hoc in our study and therefore did not affect patient management.^35^ Finally, we acknowledge that factors other than coronary atherosclerotic plaque burden should be considered in clinical decision-making regarding LLM.

### Conclusions

In this observational study of symptomatic patients undergoing CCTA evaluation for suspected CAD, the use of LLM after CCTA was associated with a lower rate of long-term adverse events among those with moderate-to-severe coronary atherosclerosis defined as PAV >5%, even in the absence of obstructive CAD. The quantification of coronary atherosclerotic plaque burden is a potential marker to guide preventive lipid-lowering therapy. Prospective studies are needed to assess its effectiveness and establish clinical decision thresholds across different software tools.

## Article Information

### Acknowledgments

Dr Maaniitty had full access to all the data in the study and takes responsibility for the integrity of the data and the accuracy of the data analysis. The data supporting the findings are not publicly available due to privacy and ethical restrictions.

### Author Contributions

Dr Maaniitty: Conceptualization, Methodology, Formal analysis, Investigation, Writing—Original Draft, Visualization, Project administration, and Funding acquisition. Dr Bär: Conceptualization, Investigation, Writing—Review and Editing. Dr Bax: Conceptualization, Investigation, Writing—Review and Editing, Supervision. Dr Knuuti: Conceptualization, Investigation, Writing—Review and Editing, Supervision, Project administration, Funding acquisition. Dr Saraste: Conceptualization, Investigation, Writing—Review and Editing, Supervision, Project administration, Funding acquisition.

### Disclosures

Dr Bär reports research grants to the institution from Medis Medical Imaging Systems, Bangerter-Rhyner Stiftung (Basel, Switzerland) and Abbott, speaker fees from Cleerly, and travel fees from Sanofi, outside the submitted work. Dr Saraste received consultancy fees from Astra Zeneca, Novo Nordisk, and Pfizer, and speaker fees from Abbott, Astra Zeneca, BMS, Janssen, and Pfizer, outside the submitted work. Dr Bax received speaker fees from Abbott, outside the submitted work. Dr Knuuti received consultancy fees from GE Healthcare and Synektik and speaker fees from Bayer, Lundbeck, Boehringer-Ingelheim, Pfizer, and Siemens, outside of the submitted work. The other authors report no conflicts.

### Supplemental Material

Tables S1–S4

Figures S1–S4

## Supplementary Material
