# Supplementary material for: Quantitative Coronary Atherosclerotic Plaque Burden From CCTA and the Benefit From Lipid-Lowering Medication
Source: Circ Cardiovasc Imaging. 2026 Apr 6;19(4):e018840. doi: 10.1161/CIRCIMAGING.125.018840 (PMC13095065; doi:10.1161/CIRCIMAGING.125.018840)
Supplement: Supplementary file 1 [file hci-19-e018840-s001.pdf]

Supplemental Material

Supplemental Figure 1. Use of lipid-lowering medication over time. An alluvial flow diagram showing the number of patients using (LLM+) and not using (LLM−) lipid-lowering medication at baseline (i.e., 6–12 months before CCTA) as well as 0–6 months and 18–24 months after CCTA, stratified by percent atheroma volume (PAV).

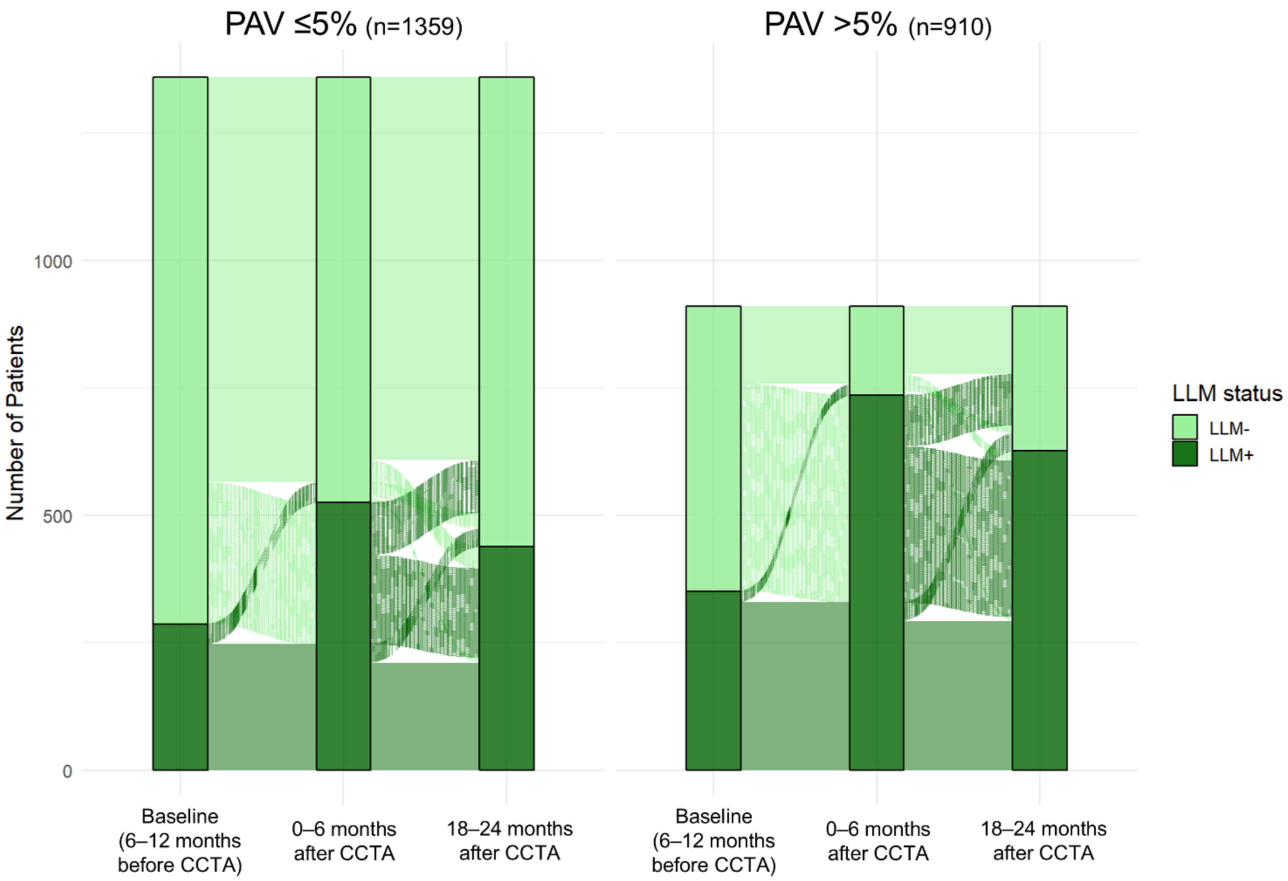

Supplemental Figure 2. Lipid-lowering medication and clinical outcome according to segment involvement score (SIS) and coronary artery calcium score (CACS). Annual (crude) incidence of death, myocardial infarction (MI), or unstable angina pectoris (UAP) in patients using (LLM+) vs. not using (LLM−) lipid-lowering medication as assessed within 6 months after CCTA, categorized by SIS of ≤4 vs. >4 (*upper panel*) and CACS of ≤100 vs. >100 (*lower panel*). CACS was available in 1862 (82%) out of 2269 patients. Error bars represent 95% confidence intervals. P-values are based on multivariable Cox regression adjusted for age, sex, smoking, diabetes, hypertension, dyslipidemia, family history, presence of typical angina, and early revascularization.

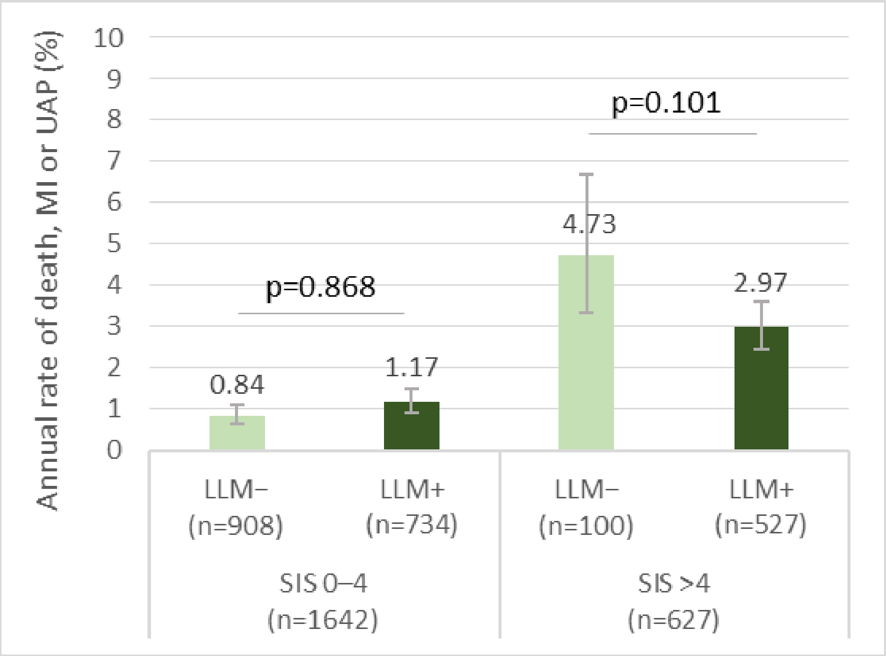

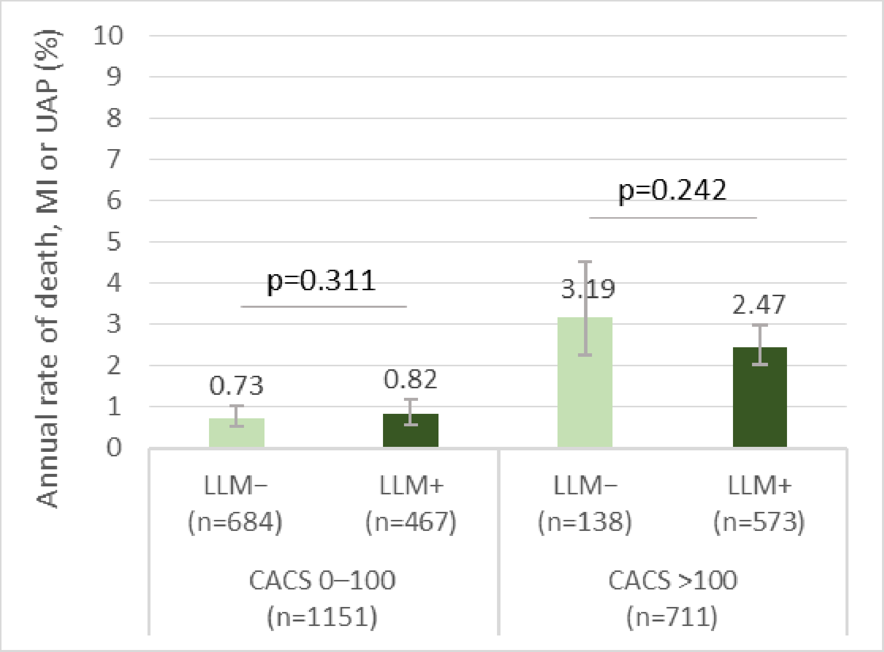

Supplemental Figure 3. Lipid-lowering medication and clinical outcome according to percent non-calcified plaque volume (NCPV). Annual (crude) incidence of death, myocardial infarction (MI), or unstable angina pectoris (UAP) in patients using (LLM+) vs. not using (LLM−) lipid-lowering medication as assessed within 6 months after CCTA, categorized by percent NCPV (≤5% vs. >5%). Error bars represent 95% confidence intervals. P-values are based on multivariable Cox regression adjusted for age, sex, smoking, diabetes, hypertension, dyslipidemia, family history, presence of typical angina, coronary artery calcium score, and early revascularization.

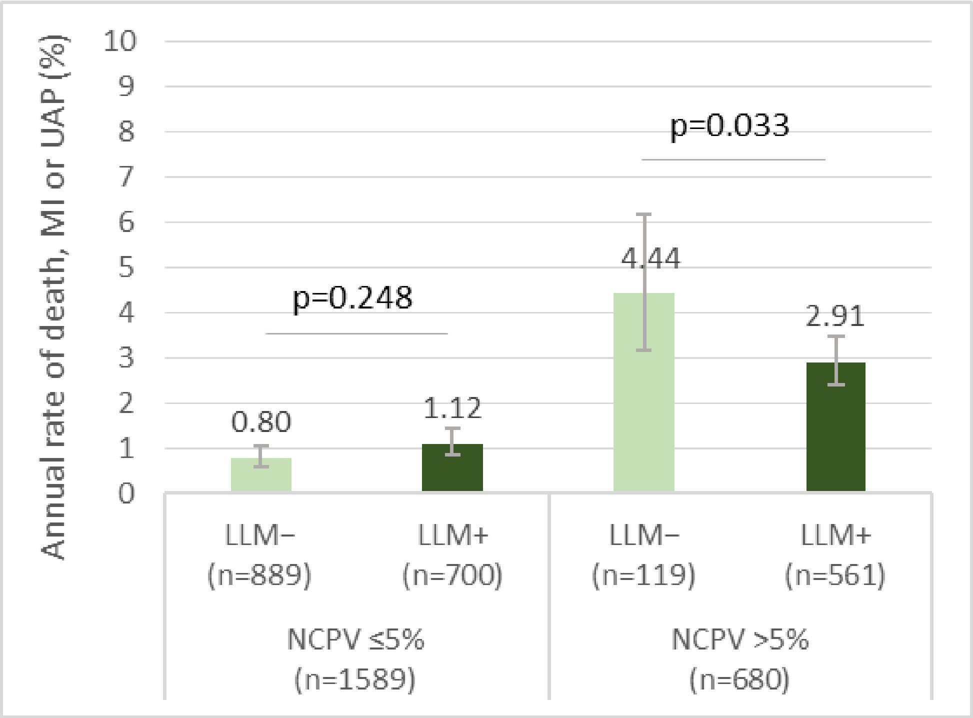

Supplemental Figure 4. Interaction of percent non-calcified plaque volume (NCPV) and lipid-lowering medication (LLM) in terms of long-term clinical outcome. Cox regression model for the interaction of continuous percent NCPV (%) and use of LLM assessed within 6 months after CCTA, for the composite endpoint of death, myocardial infarction, or unstable angina pectoris. The model is multivariable adjusted for age, sex, smoking, diabetes, hypertension, dyslipidemia, family history, presence of typical angina, and early revascularization. The solid line represents the estimate of adjusted hazard ratio (HR) of the use of LLM (vs. no LLM), with dashed lines showing 95% confidence intervals. The HR estimate equals to 1 (i.e., no effect by LLM) at percent NCPV of ~3%, and the upper 95% confidence limit equals to 1 at percent NCPV of ~6%.

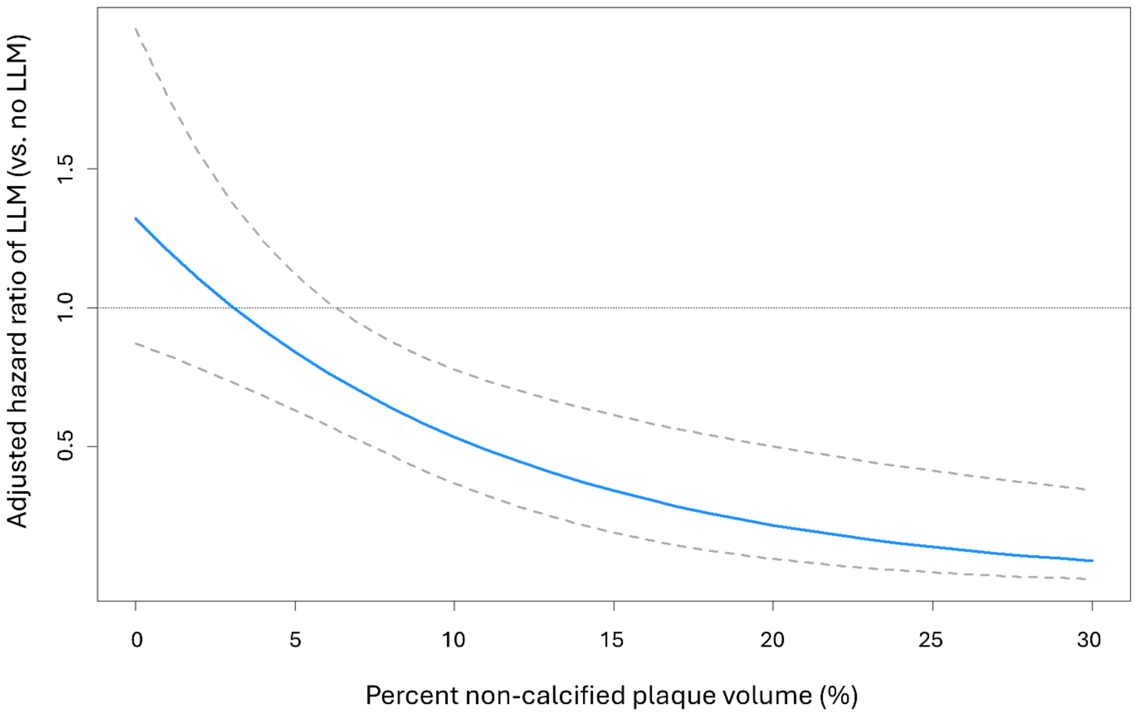

Supplemental Table 1. Multivariable binary logistic regression model for predicting the use of lipid-lowering medication within 6 months after coronary computed tomography angiography (CCTA). The model includes all recorded data available prior to CCTA, i.e., demographic and clinical data and medication prior to CCTA.

**Predictor** (n=2269)	**Odds ratio (95%CI)**	**P-value**
Clinical variables		
Age (per year)	1.06 (1.05–1.07)	<0.001
Male sex	2.36 (1.89–2.96)	<0.001
Smoking (current or previous)	1.39 (1.10–1.74)	0.005
Diabetes	1.33 (0.97–1.82)	0.076
Hypertension	1.32 (1.002–1.73)	0.049
Dyslipidemia	1.68 (1.34–2.10)	<0.001
Family history of CAD	1.30 (1.05–1.62)	0.017
Typical angina pectoris	1.39 (1.08–1.78)	0.012
Medication prior to CCTA*		
Lipid-lowering medication	9.71 (7.61–12.37)	<0.001
ACE inhibitor or ATR blocker	1.04 (0.79–1.35)	0.797
Beta blocker	1.05 (0.84–1.32)	0.647
Calcium channel blocker	0.85 (0.63–1.14)	0.271
Organic nitrate	1.22 (0.97–1.54)	0.093
Anticoagulant	1.02 (0.69–1.49)	0.933
Anti-platelet drug	0.97 (0.77–1.22)	0.808

* Data based on national registry of drug purchases assessed within 6 months prior to CCTA; with the exception of anti-platelet medication that was based on electronic medical records.

ACE = angiotensin converting enzyme, ATR = angiotensin receptor, CAD = coronary artery disease, CCTA = coronary computed tomography angiography.

Supplemental Table 2. Use of lipid-lowering medication (LLM) before and after coronary computed tomography angiography (CCTA). The use of LLM at baseline was defined based on LLM purchases at 6–12 months before CCTA whereas the use of LLM after CCTA was defined based on LLM purchases at 0–6 months after CCTA. Numbers (percentage) of patients are shown for the total study cohort and stratified by percent atheroma volume (PAV) and adverse event status at follow-up.

**Total cohort (n=2269)**			
		LLM use after CCTA	
		no	*yes*
LLM use at baseline	*no*	946 (42%)	685 (30%)
	yes	62 (3%)	576 (25%)

**PAV ≤5% (n=1359)**			
		LLM use after CCTA	
		no	*yes*
LLM use at baseline	*no*	794 (58%)	278 (20%)
	yes	40 (3%)	247 (18%)

**PAV >5% (n=910)**			
		LLM use after CCTA	
		no	*yes*
LLM use at baseline	*no*	152 (17%)	407 (45%)
	yes	22 (2%)	329 (36%)

**No composite endpoint at follow-up (n=2014)**			
		LLM use after CCTA	
		no	*yes*
LLM use at baseline	*no*	868 (43%)	602 (30%)
	yes	54 (3%)	490 (24%)

**Composite endpoint at follow-up (n=255)**			
		LLM use after CCTA	
		no	*yes*
LLM use at baseline	*no*	78 (31%)	83 (33%)
	yes	8 (3%)	86 (34%)

CCTA = coronary computed tomography angiography, LLM = lipid-lowering medication, PAV = percent atheroma volume.

Supplemental Table 3. Patient characteristics stratified by percent atheroma volume (PAV) and the use of lipid-lowering medication (LLM) within 6 months after CCTA.

	**PAV ≤5% (n=1359)**			**PAV >5% (n=910)**		
	**LLM–**	**LLM+**	**P-value**	**LLM–**	**LLM+**	**P-value**
Number	834	525		174	736	
**Clinical characteristics**						
Male sex	269	174	0.734	89	422	0.139
	32.3%	33.1%		51.1%	57.3%	
Age (years)	59 (52–65)	63 (57–69)	<0.001	68 (61–73)	67 (60–71)	0.140
Smoking (current or previous)	229	147	0.828	68	308	0.505
	27.5%	28.0%		39.1%	41.8%	
Diabetes	57	78	<0.001	38	177	0.537
	6.8%	14.9%		21.8%	24.0%	
Hypertension	351	302	<0.001	116	518	0.338
	42.1%	57.5%		66.7%	70.4%	
Dyslipidemia	391	420	<0.001	94	547	<0.001
	46.9%	80.0%		54.0%	74.3%	
Family history of CAD	396	272	0.120	61	342	0.006
	47.5%	51.8%		35.1%	46.5%	
Typical angina pectoris	147	124	0.007	40	215	0.100
	17.6%	23.6%		23.0%	29.2%	
Risk factor -weighted clinical likelihood of obstructive CAD	6% (3–10%)	10% (6–14%)	<0.001	13% (7–22%)	15% (10–25%)	0.016
**Medication prior to imaging***						
Statin or ezetimibe	114	383	<0.001	33	482	<0.001
	13.7%	73.0%		19.0%	65.5%	
ACE inhibitor or ATR blocker	249	236	<0.001	87	397	0.349
	29.9%	45.0%		50.0%	53.9%	
Beta blocker	335	272	<0.001	93	445	0.091
	40.2%	51.8%		53.4%	60.5%	
Calcium channel blocker	105	89	0.025	43	162	0.443
	12.6%	17.0%		24.7%	22.0%	
Organic nitrate	225	228	<0.001	55	337	<0.001
	27.0%	43.4%		31.6%	45.8%	
Anticoagulant	50	48	0.029	28	77	0.037
	6.0%	9.1%		16.1%	10.5%	
Anti-platelet drug	280	248	<0.001	70	403	<0.001
	33.6%	47.2%		40.2%	54.8%	
**Imaging findings**						
Coronary artery calcium score†	0 (0–8)	9 (0–58)	<0.001	226 (100–466)	377 (191–891)	<0.001
Segment involvement score	0 (0–1)	1 (0–2)	<0.001	5 (3–7)	6 (4–9)	<0.001
Obstructive (≥50%) CAD by visual CCTA reading	24	77	<0.001	73	521	<0.001
	2.9%	14.7%		42.0%	70.8%	

Percent atheroma volume	1.0% (0.4–2.1%)	2.0% (0.9–3.3%)	<0.001	9.0% (6.6–13.2%)	12.1% (7.8–20.9%)	<0.001
Percent non-calcified plaque volume	0.9% (0.4–1.8%)	1.5% (0.8–2.4%)	<0.001	5.8% (4.6–8.1%)	7.5% (5.1–10.9%)	<0.001
Percent low-density plaque volume	0.0% (0.0–0.0%)	0.0% (0.0–0.0%)	0.612	0.0% (0.0–0.0%)	0.0% (0.0–0.0%)	0.047
Percent calcified plaque volume	0.0% (0.0–0.2)	0.1% (0.0–0.8%)	<0.001	3.0% (1.4–5.8%)	4.4% (2.2–9.1%)	<0.001
Diameter stenosis degree	10% (4–17%)	16% (8–26%)	<0.001	39% (28–53%)	54% (38–71%)	<0.001
Obstructive (≥50%) CAD by AI-QCT	11	28	<0.001	55	457	<0.001
	1.3%	5.3%		31.6%	62.1%	
**Early revascularization‡**						
Early PCI	1	18	<0.001	6	152	<0.001
	0.1%	3.4%		3.4%	20.7%	
Early CABG	0	2	0.074	0	38	0.002
	0%	0.4%		0%	5.2%	
Early PCI or CABG	1	19	<0.001	6	187	<0.001
	0.1%	3.6%		3.4%	25.4%	
**Cumulative outcomes§**						
Death	28	30	0.036	35	80	<0.001
	3.4%	5.7%		20.1%	10.9%	
MI	10	3	0.247	12	43	0.600
	1.2%	0.6%		6.9%	5.8%	
UAP	3	3	0.567	3	25	0.251
	0.4%	0.6%		1.7%	3.4%	
Composite endpoint (death/MI/UAP)	39	35	0.115	47	134	0.009
	4.7%	6.7%		27.0%	18.2%	

* Data based on national registry of drug purchases assessed within 6 months prior to CCTA; with the exception of anti-platelet medication that was based on electronic medical records.
† Agatston coronary artery calcium score was measured in 1862 patients.
‡ Within 6 months after CCTA.
§ Median follow-up time 6.9 years (25th–75th percentile 4.8–9.1 years).

Categorical variables are shown as counts (percentage) and compared using Chi-square test within strata. Continuous variables are reported as median (25th–75th percentile) and compared using independent-samples Mann–Whitney U Test within strata (non-normal distribution).

ACE = angiotensin converting enzyme, AI-QCT = artificial intelligence-guided quantitative computed tomography, ATR = angiotensin receptor, CABG = coronary artery bypass grafting, CAD = coronary artery disease, CCTA = coronary computed tomography angiography, LLM = lipid-lowering medication, MI = myocardial infarction, PAV = percent atheroma volume, PCI = percutaneous coronary intervention, UAP = unstable angina pectoris.

Supplemental Table 4. Multivariable Cox regression for predicting the composite endpoint (all-cause death, myocardial infarction, unstable angina pectoris), including interaction of percent non-calcified plaque volume (NCPV, continuous variable) and use of lipid-lowering medication as assessed within 6 months after coronary computed tomography angiography (CCTA).

**Predictor (n=2269)**	**Multivariable hazard ratio (95%CI)**	**P-value**
Age (per year)	1.06 (1.04–1.08)	<0.001
Male sex	1.16 (0.88–1.54)	0.295
Smoking (current or previous)	1.45 (1.12–1.88)	0.005
Diabetes	1.09 (0.79–1.49)	0.602
Hypertension	1.25 (0.95–1.65)	0.108
Dyslipidemia	0.84 (0.64–1.10)	0.209
Family history of CAD	1.10 (0.84–1.43)	0.493
Typical angina pectoris	1.21 (0.92–1.60)	0.173
Early revascularization	0.95 (0.64–1.40)	0.787
Lipid-lowering medication (0–6 months after CCTA)	1.32 (0.87–2.00)	0.189
Percent NCPV (per percentage point)	1.17 (1.11–1.23)	<0.001
Interaction: lipid-lowering medication * percent NCPV	0.91 (0.86–0.96)	0.001

CAD = coronary artery disease, CCTA = coronary computed tomography angiography, CI = confidence intervals, NCPV = non-calcified plaque volume.
